# Monitoring of Clozapine-Induced Gastrointestinal Hypomotility

**DOI:** 10.1192/bjo.2024.342

**Published:** 2024-08-01

**Authors:** Saba Ansari, Elizabeth Spence

**Affiliations:** 1 University Hospital Monklands, Airdrie, United Kingdom; 2 Bellshill CMHT, Bellshill, United Kingdom

## Abstract

**Aims:**

Clozapine, a second-generation antipsychotic licensed for treatment-resistant schizophrenia, has a well-documented side effect profile, the most common of which is decreased gastrointestinal motility. Clozapine-induced constipation occurs more frequently than blood dyscrasias and can lead to severe complications such as paralytic ileus and intestinal blockage; in extreme cases, it can be fatal, with a fatality rate of 20–30%. The risk of gastrointestinal hypomotility is most pronounced during the initial four months of treatment; hence, weekly assessments are imperative during this period. According to Lanarkshire's local guidelines, bowel habits should be assessed at baseline, during routine blood sampling, and ideally at every clinical interaction. Our audit aims to determine the frequency of bowel habit monitoring in inpatient settings and to ascertain the prevalence of laxative prescriptions among these patients.

**Methods:**

Data were collected retrospectively from psychiatric inpatient wards in Lanarkshire for patients on Clozapine therapy. The review focused on electronic medical records to evaluate the regularity of bowel habit screening. Additionally, we examined the Hospital Electronic Prescribing and Medicines Administration (HEPMA) system to gather information on laxative prescriptions.

**Results:**

The audit revealed that bowel habit monitoring, which should be a standard practice at each clinical encounter, was found to be inconsistent. Regular assessments were documented for only 40% of patients. Monitoring was most thorough in rehabilitation wards, where patients on Clozapine had their gastrointestinal function assessed routinely through screening questionnaires. Furthermore, 80% of the surveyed patient population was documented as having been prescribed laxatives.

**Conclusion:**

The documentation of bowel movements for inpatients on Clozapine was suboptimal, leading to the potential oversight of critical side effects. The audit highlights a discrepancy in adherence to national and Lanarkshire's local guidelines for the monitoring of inpatients treated with Clozapine. To rectify this, we recommend the implementation of a standardized screening protocol to assess constipation risk systematically. Proactive monitoring should be incorporated into regular clinical evaluations for patients on Clozapine, ensuring that this assessment occurs at every clinical interaction. This approach is crucial not only for patient safety but also for enhancing treatment efficacy and patient quality of life. Moreover, these measures will likely lead to improved documentation and compliance with established guidelines, thereby reducing the incidence of preventable complications associated with Clozapine-induced constipation.

